# A Hexabenzocoronene‐Based Helical Nanographene

**DOI:** 10.1002/chem.202001471

**Published:** 2020-07-10

**Authors:** Max M. Martin, Frank Hampel, Norbert Jux

**Affiliations:** ^1^ Department of Chemistry and Pharmacy & Interdisciplinary Center for, Molecular Materials (ICMM) Organic Chemistry II Friedrich-Alexander-University Erlangen-Nuernberg Nikolaus-Fiebiger-Str. 10 91058 Erlangen Germany

**Keywords:** carbon allotrope, helicene, hexabenzocoronene, nanographene, polycyclic aromatic hydrocarbons

## Abstract

A synthetic route towards a novel hexabenzocoronene‐based helical nanographene motif was developed. A hexaphenylbenzene precursor was therefore designed, which cannot undergo, due to steric restrictions, a complete planarization reaction. This precursor was transformed under oxidative cyclodehydrogenation conditions to a π‐extended [5]helicene, which was fully characterized including X‐ray diffraction analysis.

## Graphical abstract

**A twisted nanographene** that is based on the well‐established hexabenzocoronene motif is presented. A [5]helical unit was implemented into the nanographene structure with the help of steric repulsive effects. The π‐extended helicene was characterized by UV/Vis absorption and emission spectroscopy as well as X‐ray crystallography.

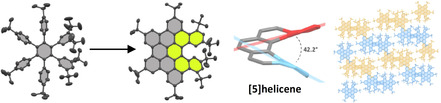

The first preparation of buckminsterfullerenes that was reported 35 years ago (1985)^1^ marked the beginning of the era of synthetic carbon allotropes.^2^ In the following years, intense carbon‐based material research was conducted that resulted in the discovery of two more members of the carbon family: carbon nanotubes^3^ in 1991 and graphene^4^ in 2004. The aim to explore and discover new compositions of pure carbon is still unabated. Only recently, the cyclic sp‐hybridized cyclo[18]carbon molecule^5^ was visualized on a surface and confirmed as a member of the carbon allotrope family.^6^ Additionally, hypothetical carbon‐only architectures, such as helical or twisted derivatives of graphene, toroidal carbon nanotubes or Mackay crystals, are subject of ongoing research.^7^ Important steps towards elusive novel carbon‐only materials were already achieved by the synthesis of model compounds that possess key characteristics of new carbon allotropes. Especially, polycyclic aromatic hydrocarbons (PAHs) and nanographenes with a non‐planar, twisted^7^, ^8^, ^9^, ^10^, ^11^, ^12^, ^13^ or helical^14^, ^15^, ^16^, ^17^, ^18^, ^19^, ^20^, ^21^, ^22^, ^23^ topology are becoming increasingly popular in the field of carbon‐based materials.^24^ Due to the out‐of‐plane distortion, a third dimension is introduced to the nanographenes that results in unique properties different to the ones of the planar analogues. Herein, we would like to extend the synthetic toolbox of non‐planar nanographenes by a simple and efficient route that is based on well‐established reactions used for hexa‐*peri*‐hexabenzocoronene (HBC) type chemistry. Precise control over the formation of the helical unit should be gained by the help of steric repulsive effects that prevent complete planarization during the final oxidative cyclodehydrogenation reaction step. This strategy and all synthetic details are presented in the following paper.

First, a suitable tolane, which is capable of forming a helical HBC‐based product in the upcoming reaction steps, had to be designed. For that, tetra‐*tert*‐butyl‐tolane **4** was selected. Although a synthesis of **4** is already literature known,^25^ we employed a different approach, which easily allows to prepare gram quantities of tolane **4**. With this procedure, no precious reagents or expensive purification techniques, for example, column chromatography, were necessary. At first, 3,5‐di‐*tert*‐butylbenzaldehyde was reacted under McMurry conditions^26^ to stilbene **2** (Scheme 1) using zinc powder as the reducing agent and TiCl_4_ as the titanium species. After aqueous workup and recrystallization from CH_2_Cl_2_/MeOH, product **2** was obtained in suitable yields of 69 %. Stilbene **2** was subsequently brominated^27^ using Br_2_, which yielded the double brominated compound **3** after aqueous workup in almost quantitative yields (95 %). Next, a double HBr elimination^27^ under strong basic conditions (KO*t*Bu in THF) generated the desired tolane **4**, which was isolated after aqueous workup and recrystallization from CH_2_Cl_2_/MeOH in 78 % yield. With tolane **4** and tetracyclone **5** a standard [4+2] Diels–Alder reaction was performed. The reaction proceeded smoothly at 260 °C in the microwave reactor, which yielded hexaphenylbenzene (HPB) **6** after recrystallization in 81 % yield. Finally, an oxidative cyclodehydrogenation reaction was applied to form an extended aromatic π‐system. However, due to the steric demand of the *tert*‐butyl groups attached to the phenyl rings drawn in red (Scheme 1), it seemed impossible to form all six C−C bonds as it is usually the case for HBCs and related materials. As a result, the twisted nanographene **7**, in which five C−C bonds were formed, was generated under standard reaction conditions (FeCl_3_, CH_3_NO_2_/CH_2_Cl_2_) and isolated in 80 % yield. The reaction proceeded smoothly without any side product formation (followed by TLC).

**Scheme 1:**
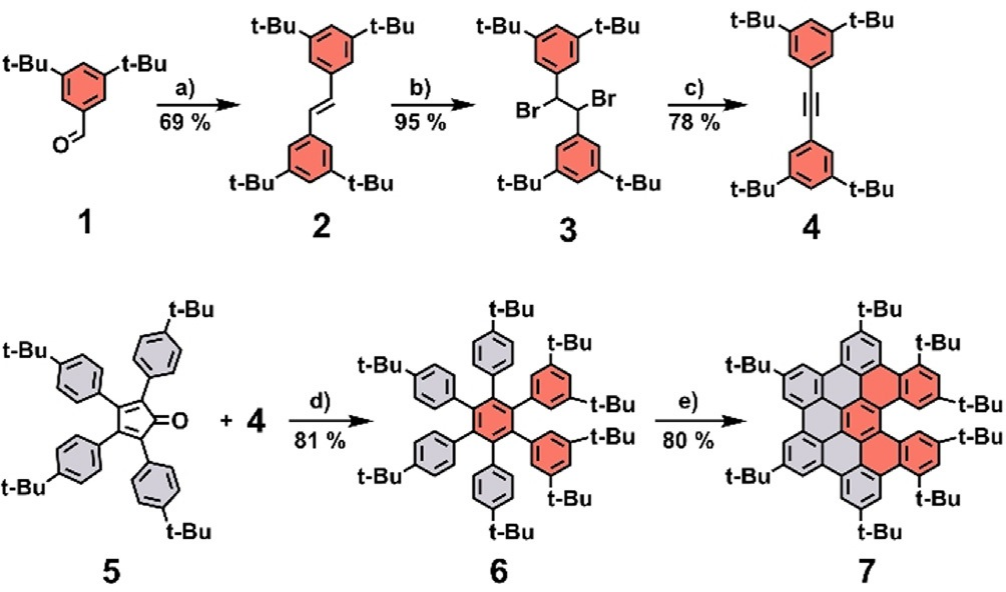
Synthesis of HBC‐based [5]helicene **7**. a) Zn, TiCl_4_, THF, 70 °C, 22 h; b) Br_2_, CHCl_3_, rt, 30 min; c) KO*t*Bu, THF, 0 °C, 20 min; d) Ph_2_O, 260 °C in microwave reactor, 12 h; e) FeCl_3_, CH_3_NO_2_, CH_2_Cl_2_, 0 °C, 100 min.

Product **7** as well as all of the precursor compounds were carefully characterized by NMR spectroscopic and mass spectrometric techniques (for details see Supporting Information). X‐ray crystallography of **2**, **4**, **6** and **7** confirmed furthermore the successful synthesis of these compounds and allowed to get an insight into the solid‐state behavior. Generally, molecules within this project showed a high tendency to form crystalline structures, as some of the single crystals, for example, the ones of **7**, grew within 24 h. The structures of **2**, **4** and **6** are depicted in the Supporting Information (Figures S1–S4) and a detailed structural analysis of the target compound **7** is shown in Figure 1. HBC‐based [5]helicene **7** crystallizes in a monoclinic crystal system with the space group *P*2_1_/*n*. The helical character of **7**, which is defined by the sum of torsion angles (81.5°) and the interplanar angle (42.2°),^28^ was unambiguously confirmed by the crystal structure. The helicity values of **7** significantly differ to the ones of previously reported [5]helicenes, for example, to a carbon[5]helicene (sum of torsion angles: 65.9–67.8°; interplanar angle: 47.3–51.3°).^29^, ^30^ For more details see Tables S6 and S7 in the Supporting Information. This deviation can be explained by two major structural variations that affect the helical properties of the HBC‐based [5]helicene. On the one hand, the rigid aromatic backbone of **7** (Figure 1 a, drawn in grey) reduces the flexibility, especially of the inner rings B, C and D, and therefore limits the overall twisting capability of the helix. However, on the other hand, the bulky *tert*‐butyl groups counteract, due to steric repulsion, to the effects of the stiff backbone and are leading to an increased distortion of the helical unit, particularly of the outer rings A and E. The sum of these two effects lead to a helical unit with an unevenly distributed helical character with areas of in‐ and decreased degree of distortion. This is also supported by the large bond length variations of 1.41–1.48 Å between the inner helical carbon atoms (Figure 1 b). The crystal packing (Figure 1 d,e) reveals that both enantiomers (*M*) and (*P*) are present, therefore forming a racemic crystal. The molecules are, compared to the crystal packing of, for example, hexa*‐tert*‐butyl‐HBC^31^ relatively loosely packed with distances of ≥5 Å between the aromatic planes of the molecules. Hence, no π–π interactions and only CH–π as well as London dispersion interactions were found, which seem to be the driving force for the preferential formation of all shown structures in the crystal. Interestingly, layers of a single chirality are found in the crystal of **7**, generating an alternating pattern of (*M*) and (*P*) enantiomer layers (compare Figure S5).

**Figure 1:**
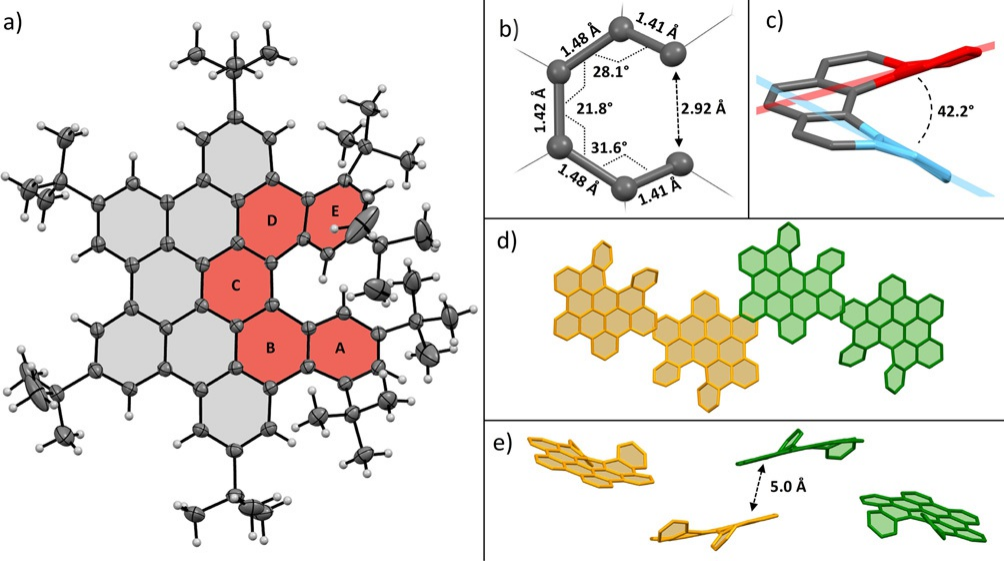
a) Crystal structure of HBC‐based [5]helicene **7**. Structure is depicted as ORTEP model with thermal ellipsoids drawn at a 50 % probability level; b) torsion angles and bond lengths of the inner helix; c) determination of the interplanar angle; d), e) packing motif; hydrogen atoms, *tert*‐butyl groups and solvent molecules are omitted for clarity. Helicenes with the same chirality are drawn in the same color. CCDC: 1990061; see Experimental Section for details.

The differences in the spectroscopic properties between the HBC‐based [5]helicene **7** and a planar reference compound, hexa‐*tert*‐butyl‐HBC,^32^ were studied by UV/Vis absorption and emission spectroscopy. The UV/Vis absorption spectrum of **7** (Figure 2 a) features a redshifted maximum at 367 nm (*p*‐band) with a significantly decreased molar extinction coefficient compared to the reference HBC. Furthermore, a reduced resolution of the fine structure, due to a general broadening of all absorption bands, was observed. This is also true for the fluorescence spectrum (Figure 2 b), which shows, aside from broadened signals, hypsochromically shifted emission maxima at 475 and 506 nm for **7**. Therefore, the implementation of a helical unit into a nanographene has clear effects on the photophysical properties, such as on the position but also on the shape of the respective absorption and emission bands.

**Figure 2:**
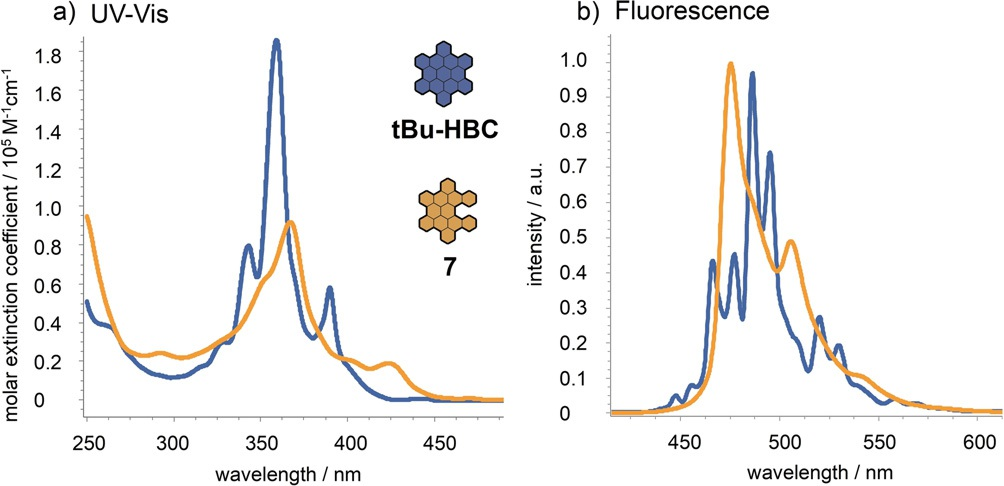
a) UV/Vis absorption spectra of HBC‐based [5]helicene **7** and reference compound hexa‐*tert*‐butyl‐HBC^34^ in CH_2_Cl_2_; b) normalized steady state fluorescence emission spectra upon irradiation of the absorption maximum.

In conclusion, we have presented a new procedure for the synthesis of a HBC‐based twisted nanographene that contains a [5]helical unit. The helicity was introduced with the help of sterically demanding groups that prevent a complete closure to planar PAHs during the oxidative cyclodehydrogenation step. These results complement our previous findings, in which related nitrogen‐doped HBC‐like [5]helicenes were formed, however, controlled by electronic effects.^33^ The chiral separation and characterization as well as the usage of the twisted nanographene motif of **7** as a platform for further functionalized hybrid materials, for example, analogous architectures to our previously reported porphyrin‐HBCs,^34^, ^35^, ^36^, ^37^, ^38^, ^39^, ^40^, ^41^, ^42^ are currently under investigation.

## Experimental Section

**Crystallographic data**: Deposition number 1990061 contains the supplementary crystallographic data for this paper. These data are provided free of charge by the joint Cambridge Crystallographic Data Centre and Fachinformationszentrum Karlsruhe Access Structures service.

## Conflict of interest

The authors declare no conflict of interest.

## Supporting information

As a service to our authors and readers, this journal provides supporting information supplied by the authors. Such materials are peer reviewed and may be re‐organized for online delivery, but are not copy‐edited or typeset. Technical support issues arising from supporting information (other than missing files) should be addressed to the authors.

Supplementary
