# Supplementary material for: A Hexabenzocoronene‐Based Helical Nanographene
Source: Chemistry. 2020 Jul 10;26(45):10210–2. doi: 10.1002/chem.202001471 (PMC7496628; doi:10.1002/chem.202001471)
Supplement: Supplementary file 1 — Supplementary [file CHEM-26-10210-s001.pdf]

Chemistry–A European Journal

Supporting Information

A Hexabenzocoronene-Based Helical Nanographene

Max M. Martin, Frank Hampel, and Norbert Jux*[a]

Table of Content

1 General Information ... 2
2 Synthetic Procedures ... 3
3 X-ray Crystallography ... 7
4 Comparison to Literature Known [5]Helicenes ... 13
5 Spectral Appendix ... 14
6 References ... 19

1 General Information

All chemicals were purchased from Sigma-Aldrich and used without any further purification. Solvents were distilled prior to usage. Dichloromethane and chloroform were neutralized with K_2CO_3 before distillation. Thin layer chromatography (TLC) was performed on Merck silica gel 60 F524, detected by UV-light (254 nm, 366 nm). Column chromatography and flash column chromatography were performed on Macherey-Nagel silica gel 60 M (deactivated, 230-400 mesh, 0.04–0.063 mm). NMR spectroscopy was performed on a Bruker Avance 400 (^{1}H: 400 MHz, ^{13}C: 101 MHz) or Bruker Avance Neo Cryo-Probe DCH (^{1}H: 600 MHz, ^{13}C: 150 MHz). Deuterated solvents were purchased from Sigma Aldrich and used as received. Chemical shifts are referenced to residual protic impurities in the solvents (^{1}H: $CHCl_3$: 7.24 ppm) or the deuterated solvent itself (^{13}C: $CDCl_3$: 77.0 ppm). The resonance multiplicities are indicated as "s" (singlet), "d" (doublet), "t" (triplet), "q" (quartet) and "m" (multiplet). High resolution mass spectrometry was performed either on a LDI/MALDI-ToF Bruker Ultraflex Extreme machine or on a APPI-ToF mass spectrometer Bruker maXis 4G UHR MS/MS spectrometer. In case of MALDI, *trans*-2-[3-(4-*tert*-butylphenyl)-2-methyl-2-propenylidene]malononitrile (DCTB) was used as the matrix. Microwave reactions were carried out in a mono-mode microwave reactor Biotage Initiator$^+$. The microwave assisted reactions were carried out exclusively in the fixed hold time mode using an external IR temperature sensor. UV/Vis spectroscopy was carried out on a Varian Cary 5000 UV-Vis-NIR spectrometer.

2 Synthetic Procedures

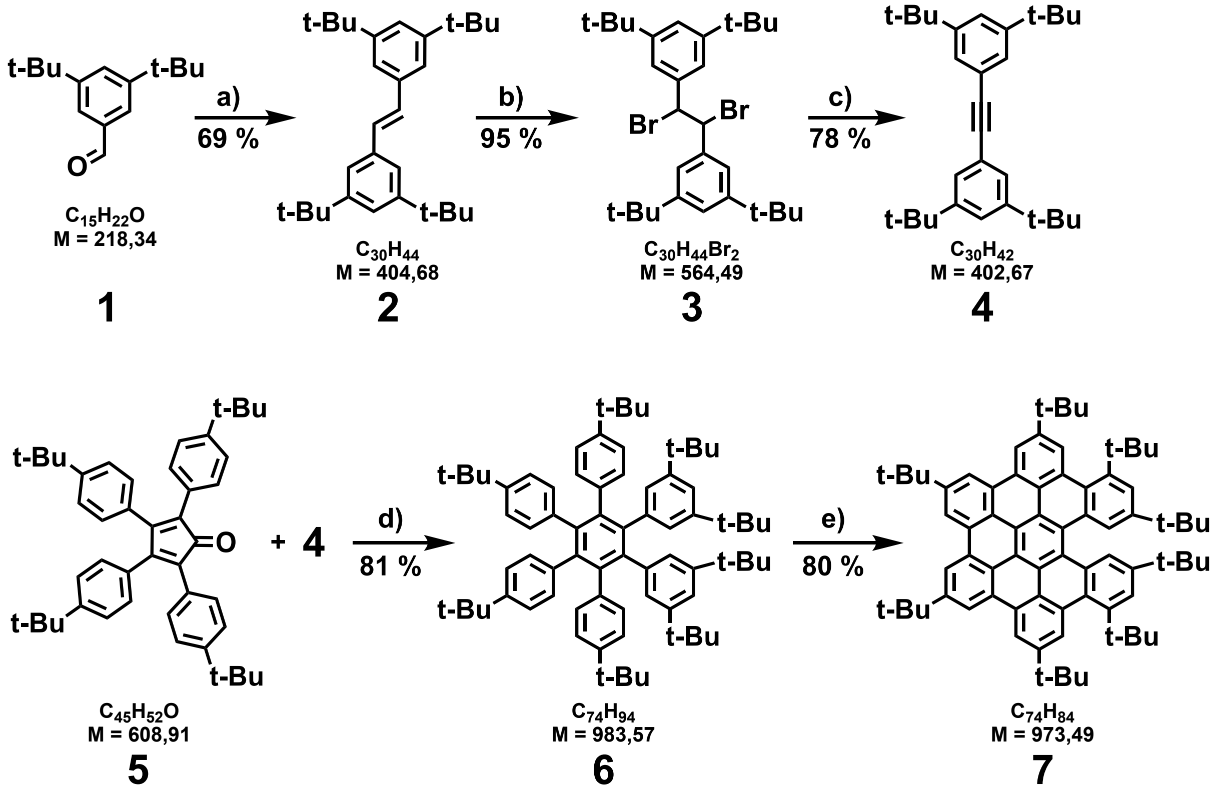

Scheme S1. Synthesis of [5]helicene-HBC **7**. a) Zn, $TiCl_4$, THF, 70 °C; b) Br_2, $CHCl_3$; c) KOtBu, THF, 0 °C; d) Ph_2O, 260 °C, 12 h; e) $FeCl_3$, CH_3NO_2, CH_2Cl_2, 0 °C.

Tert-butyl substituted tetracyclone **5** was prepared according to the literature.[1-2]

1,2-Bis(3,5-di-*tert*-butylphenyl)ethylene 2
Modified McMurry conditions from Jeong et al.[3]

A 500 mL Schlenk flask, charged with zinc powder (9.95 g, 151 mmol, 5.6 equiv), was flame dried under vacuum and put under N_2 atmosphere. Dry, degassed THF (150 mL) was added and the suspension was cooled with an ice/NaCl bath. $TiCl_4$ (8.25 mL, 75.5 mmol, 2.8 equiv) was added slowly (caution: heavy reaction). The mixture was heated to 70 °C and 3,5-di-*tert*-butylbenzaldehyde, dissolved in dry THF (125 mL), was added via a dropping funnel. After complete addition, the reaction was heated to 70 °C for 22 h under N_2 atmosphere. H_2O (100 mL) was added, the flask was cooled with an ice bath and 10 % HCl (100 mL) was added until the remaining zinc dissolves. The product was extracted with CH_2Cl_2 (2 x 250 mL) and the organic phase was washed with sat. $NaHCO_3$ (400 mL), H_2O (400 mL) and dried over $MgSO_4$. The solvent was removed, the product dissolved in little CH_2Cl_2 and precipitated via the addition of MeOH. The product was obtained as a white solid in 69.3 % yield (3.78 g, 9.35 mmol).

^{1}H NMR (300 MHz, $CDCl_3$) δ 7.37 (d, *J* = 1.8 Hz, 4H), 7.33 (t, *J* = 1.8 Hz, 2H), 7.12 (s, 2H), 1.36 (s, 36H).

^{13}C NMR (101 MHz, $CDCl_3$) δ 150.93, 136.78, 129.03, 121.85, 120.80, 34.87, 31.50.

HRMS (APPI, MeCN/MeOH) for $C_{30}H_{44}(M^+)$, calc.: 404.3438, found: 404.3439.

The product was identified as the *E*-isomer (comparison with the literature).[4-6]

Bromination

1,2 Bis(3,5-di-*tert*-butylphenyl)ethylene **2** (3.76 g, 9.28 mmol, 1 equiv) was dissolved in $CHCl_3$ (45 mL). Br_2 (1.56 g, 0.50 mL, 9.75 mmol, 1.05 equiv), diluted with $CHCl_3$ (5 mL) was added slowly via syringe. The reaction progress was followed via TLC (hexanes/CH_2Cl_2 – 4:1). After 30 min all starting material was consumed and the reaction was quenched with 10 % $Na_2S_2O_3$ (50 mL). The organic phase was extracted with CH_2Cl_2, washed with H_2O (50 mL) and dried over $MgSO_4$. Product **3** was obtained in 95.6 % yield (5.01 g, 8.87 mmol) as a mixture of two isomers in a ratio of 1.0 : 1.6 (62 % and 38 %). Separation of the two isomers was not necessary as in the following elimination step both isomers yield the same product.

^{1}H NMR (400 MHz, $CDCl_3$) δ 7.11 (t, *J* = 1.8 Hz, 2H), 6.91 (d, *J* = 1.8 Hz, 4H), 5.37 (s, 2H), 1.15 (s, 36H). Major isomer.

^{1}H NMR (400 MHz, $CDCl_3$) δ 7.38 (t, *J* = 1.8 Hz, 2H), 7.31 (d, *J* = 1.8 Hz, 4H), 5.49 (s, 2H), 1.35 (s, 36H). Minor Isomer

^{13}C NMR (101 MHz, $CDCl_3$) δ 150.92, 150.62, 139.04, 137.73, 122.78, 122.32, 122.18, 61.63, 57.71, 34.93, 34.67, 31.46, 31.31. Signal set of both isomers.

HRMS (APPI, toluene): not detectable.

Spectroscopic data in good agreement with the literature.[7]

Tetra-*tert*-butyl-tolane 4

3 (4.98 g, 8.82 mmol, 1 equiv) was dissolved in THF (100 mL) and cooled with an ice bath. Potassium-*tert*-butoxide (2.97 g, 26.5 mmol, 3 equiv) was added in portions as a solid and the mixture was stirred for 10 min under ice bath cooling. The ice bath was removed and the reaction was stirred for further 10 min before H_2O (100 mL) was added. The product was extracted with CH_2Cl_2 (3 x 50 mL), the organic phase was washed with H_2O (100 mL) and dried over $MgSO_4$. The product was dissolved in little

CH_2Cl_2 (≈ 20 mL) and precipitated via the addition of MeOH. The pure product was obtained as a white solid in 78.3 % yield (2.78 g, 6.91 mmol).

^{1}H NMR (400 MHz, $CDCl_3$) δ 7.39 (d, *J* = 1.8 Hz, 4H), 7.38 (t, *J* = 1.7 Hz, 2H), 1.33 (s, 36H).

^{13}C NMR (101 MHz, $CDCl_3$) δ 150.76, 125.89, 122.54, 122.41, 89.22, 34.83, 31.36.

HRMS (APPI, toluene) for $C_{30}H_{42}$(M^+), calc.: 402.3281, found: 402.3290.

Spectroscopic data in good agreement with the literature.[8-9]

Octa-*tert*-butyl-HPB 6

Modified Diels-Alder conditions from Martin et al.[1]

Tetra-*tert*-butyl-tolane **4** (100 mg, 248 μmol, 1 equiv) and *tert*-butyl-tetracyclone **5** (151 mg, 248 μmol, 1 equiv) were dissolved in Ph_2O (1.0 mL) and heated in the microwave reactor to 260 °C for 12 h. The reaction mixture was diluted with little CH_2Cl_2 and the product precipitated via the addition of MeOH. The product was filtered off, washed with MeOH and recrystallized once more from CH_2Cl_2/MeOH. The pure product was obtained as a white solid in 81.0 % yield (198 mg, 201 μmol).

^{1}H NMR (400 MHz, $CDCl_3$) δ 6.81 – 6.75 (m, 10H), 6.68 – 6.59 (m, 12H), 1.08 (s, 18H), 1.08 (s, 18H), 0.89 (s, 36H).

^{13}C NMR (101 MHz, $CDCl_3$) δ 148.34, 147.31, 147.13, 140.88, 140.52, 140.45, 139.43, 138.24, 138.14, 131.23, 126.99, 123.18, 122.96, 117.96, 34.24, 34.03, 34.00, 31.28, 31.24, 31.20.

HRMS (MALDI, dctb) for $C_{74}H_{94}$ (M^+), calc.: 982.7350, found: 982.7373.

Note: No attempts to further optimize our previously reported Diels-Alder reaction conditions[1] (*e.g.* shorter reaction time) were tested at this point.

HBC-based-[5]-helicene 7

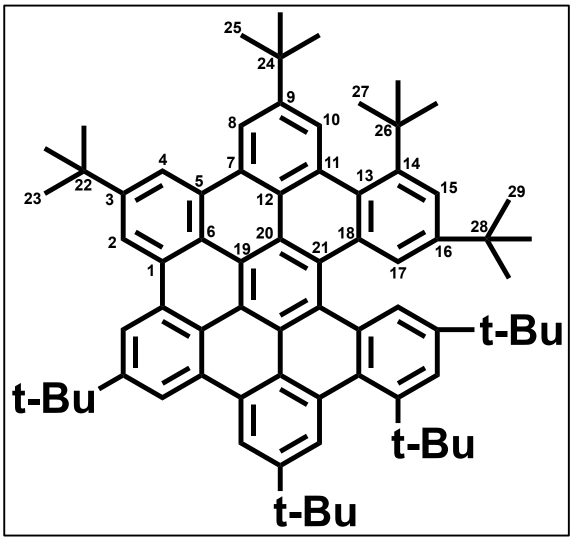

Octa-*tert*-butyl-HPB **6** (50 mg, 50.8 μmol, 1 equiv) was dissolved in CH_2Cl_2 (50 mL), cooled with an ice bath and degassed (20 min N_2 bubbling through the solution). Dry $FeCl_3$ (164 mg, 1.02 mmol, 20 equiv) dissolved in CH_3NO_2 (0.4 mL) was added to the reaction and the flow of N_2 through the solution was maintained for further 20 min. The reaction was stirred under ice bath cooling for 80 min and quenched via the addition of MeOH (15 mL). The crude was purified via silica plug filtration (hexanes/CH_2Cl_2 – 3:1, ∅ 3 cm · 6 cm) followed by recrystallization from CH_2Cl_2/MeOH. The product was obtained as a yellow solid in 80.1 % yield (39.6 mg, 40.7 μmol).

^{1}H NMR (601 MHz, $CDCl_3$) δ 9.23 – 9.19 (m, 2H, H-2), 9.19 – 9.16 (m, 2H, H-4), 9.06 – 9.02 (m, 2H, H-8), 8.73 (d, *J* = 1.8 Hz, 2H, H-10), 7.82 (d, *J* = 2.1 Hz, 2H, H-15), 7.75 (d, *J* = 2.1 Hz, 2H, H-17), 1.84 (s, 18H, H-27), 1.79 (s, 18H, H-23), 1.72 (s, 18H, H-25), 1.12 (s, 18H, H-29).

^{13}C NMR (151 MHz, $CDCl_3$) δ 148.68 (C-3), 147.19 (C-14), 146.31 (C-9), 144.94 (C-16), 133.49 (C-18), 131.49 (C-11), 130.59 (C-5), 130.31 (C-1), 130.20 (C-13), 129.45 (C-7), 128.16 (C-10), 126.32 (C-17), 125.81 (C-15), 125.56 (C-21), 123.58 (C-6), 123.12 (C-12), 122.81 (C-20), 119.79 (C-19), 118.77 (C-4), 118.36 (C-2), 118.02 (C-8), 38.32 (C-26), 35.82 (C-24), 35.70 (C-22), 34.18 (C-28), 34.16 (C-27), 32.04 (C-25), 32.03 (C-23), 31.27 (C-29).

HRMS (MALDI, dctb) for $C_{74}H_{84}$ (M^+), calc.: 972.6568, found: 972.6563.

UV/Vis (CH_2Cl_2): λ [nm] (ε [$M^{-1}cm^{-1}$]) = 292 (24300), 367 (91700), 424 (18900).

Fluorescence (CH_2Cl_2): $\lambda_{exc.}$ [nm] = 367, **$\lambda_{emission}$ [nm] (rel. int.)** = 475 (1.00), 506 (0.49).

3 X-ray Crystallography

1,2-Bis(3,5-di-*tert*-butylphenyl)ethylene 2

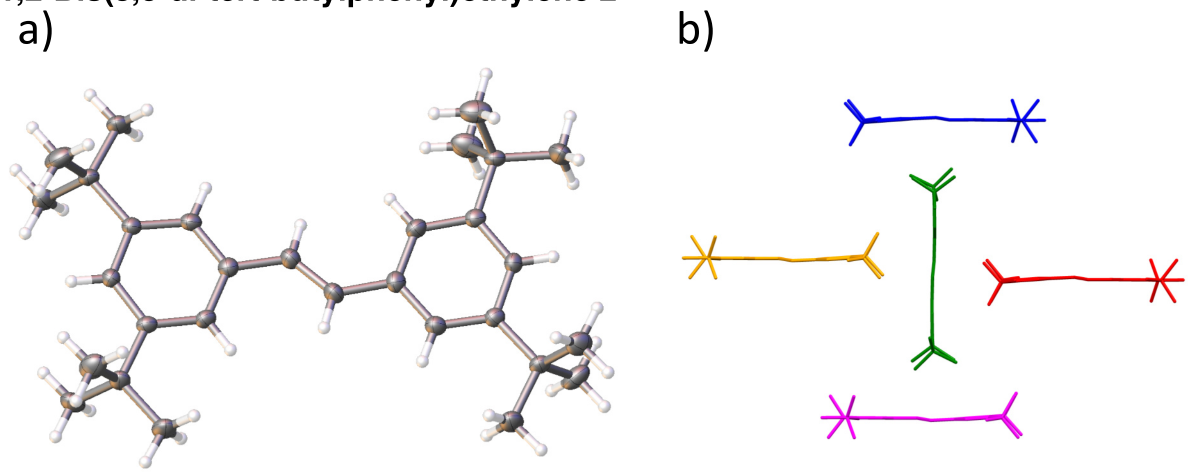

Figure S1. Crystal structure of 1,2-bis(3,5-di-*tert*-butylphenyl)ethylene **2**. a) ORTEP representation with thermal ellipsoids drawn at 50 % probability; b) packing motif. CCDC 1990059.

Table S1. Crystal data and structure refinement for 18Jux_MM02.

Identification code	18Jux_MM02
Empirical formula	$C_{30}H_{44}$
Formula weight	404.65
Temperature/K	153.05(10)
Crystal system	triclinic
Space group	P-1
a/Å	10.1377(6)
b/Å	12.8339(8)
c/Å	16.7848(13)
α/°	100.517(6)
β/°	95.164(6)
γ/°	110.471(6)
Volume/$Å^3$	1983.3(2)
Z	3
$\rho_{calc}g/cm^3$	1.016
μ/mm^{-1}	0.412
F(000)	672.0
Crystal size/mm^3	0.162 × 0.069 × 0.054
Radiation	CuKα (λ = 1.54184)
2Θ range for data collection/°	7.554 to 123.12
Index ranges	-6 ≤ h ≤ 11, -14 ≤ k ≤ 10, -18 ≤ l ≤ [illegible]
Reflections collected	8860
Independent reflections	5884 [R_{int} = 0.0529, R_{sigma} = 0.0691]
Data/restraints/parameters	5884/0/424
Goodness-of-fit on F^2	1.069
Final R indexes [I>=2σ (I)]	R_1 = 0.0655, wR_2 = 0.1723
Final R indexes [all data]	R_1 = 0.1031, wR_2 = 0.1951
Largest diff. peak/hole / e $Å^{-3}$	0.37/-0.35
CCDC No.	1990059

Tetra-*tert*-butyl-tolane 4 (structure 1)

a) b)

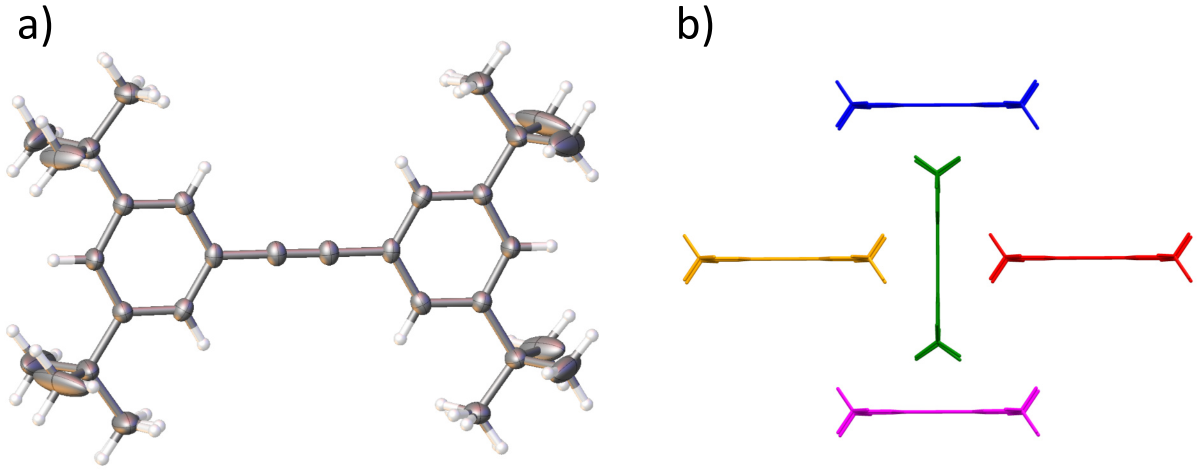

Figure S2. Crystal structure of tetra-*tert*-butyl-tolane **4**. a) ORTEP representation with thermal ellipsoids drawn at 50 % probability; b) packing motif. Crystals were grown in a CH_2Cl_2/MeOH solvent mixture. CCDC 1990058.

Table S2. Crystal data and structure refinement for 17Jux_MM12.

Identification code	17Jux_MM12
Empirical formula	$C_{30}H_{42}$
Formula weight	402.63
Temperature/K	154(4)
Crystal system	triclinic
Space group	P-1
a/Å	6.5943(6)
b/Å	9.9805(10)
c/Å	10.0954(10)
α/°	86.901(8)
β/°	80.210(8)
γ/°	73.847(8)
Volume/Å^3	628.88(11)
Z	1
ρ_{calc}g/cm^3	1.063
μ/mm^{-1}	0.433
F(000)	222.0
Crystal size/mm^3	0.338 × 0.219 × 0.109
Radiation	CuKα (λ = 1.54184)
2Θ range for data collection/°	8.89 to 145.958
Index ranges	-7 ≤ h ≤ 7, -8 ≤ k ≤ 12, -12 ≤ l ≤ 11
Reflections collected	3576
Independent reflections	2358 [R_{int} = 0.0226, R_{sigma} = 0.0285]
Data/restraints/parameters	2358/0/142
Goodness-of-fit on F^2	1.030
Final R indexes [I>=2σ (I)]	R_1 = 0.0432, wR_2 = 0.1167
Final R indexes [all data]	R_1 = 0.0522, wR_2 = 0.1274
Largest diff. peak/hole / e Å^{-3}	0.16/-0.26
CCDC No.	1990058

Tetra-*tert*-butyl-tolane 4 (structure 2)

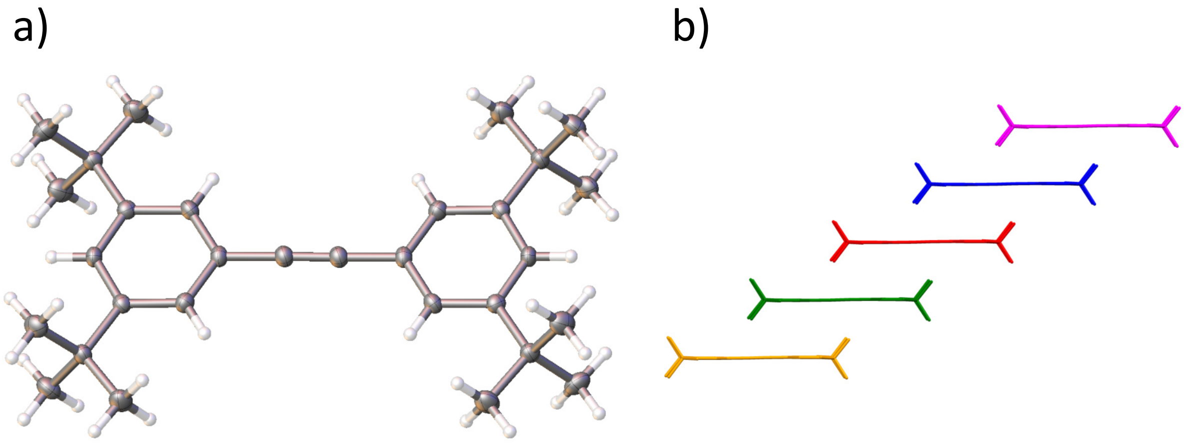

Figure S3. Second crystal structure of tetra-*tert*-butyl-tolane **4**. a) ORTEP representation with thermal ellipsoids drawn at 50 % probability; b) packing motif. Crystals were grown in a CH_2Cl_2/MeOH solvent mixture. CCDC 1990057.

Table S3. Crystal data and structure refinement for 17Jux_MM03.

Identification code	17Jux_MM03
Empirical formula	$C_{30}H_{42}$
Formula weight	402.63
Temperature/K	153.05(10)
Crystal system	monoclinic
Space group	I2/m
a/Å	10.1667(3)
b/Å	17.8848(4)
c/Å	16.1287(4)
α/°	90
β/°	97.769(2)
γ/°	90
Volume/Å^3	2905.75(13)
Z	4
ρ_{calc}g/cm^3	0.920
μ/mm^{-1}	0.375
F(000)	888.0
Crystal size/mm^3	0.287 × 0.108 × 0.082
Radiation	CuKα (λ = 1.54184)
2Θ range for data collection/°	7.418 to 145.688
Index ranges	-12 ≤ h ≤ 10, -21 ≤ k ≤ 21, -19 ≤ l ≤ 17
Reflections collected	8463
Independent reflections	2942 [R_{int} = 0.0245, R_{sigma} = 0.0239]
Data/restraints/parameters	2942/15/161
Goodness-of-fit on F^2	1.096
Final R indexes [I>=2σ (I)]	R_1 = 0.0865, wR_2 = 0.2435
Final R indexes [all data]	R_1 = 0.0965, wR_2 = 0.2514
Largest diff. peak/hole / e Å^{-3}	0.40/-0.40
CCDC No.	1990057

Octa-*tert*-butyl-HPB 6

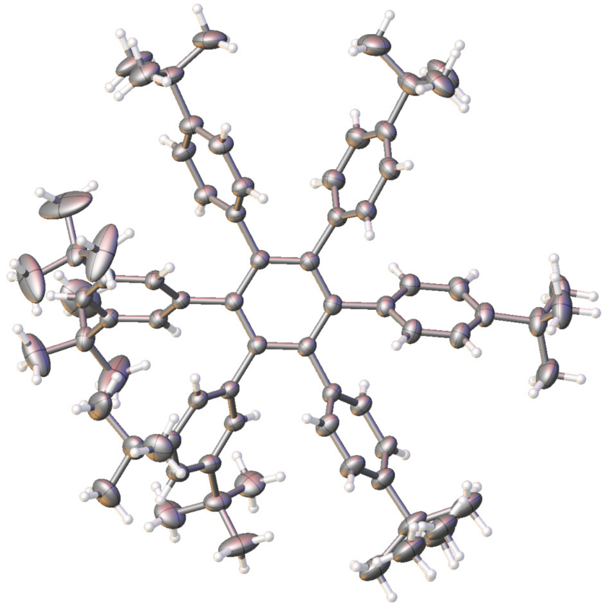

Figure S4. Crystal structure of octa-*tert*-butyl-HPB **6**. ORTEP representation with thermal ellipsoids drawn at 50 % probability. Crystals were grown in CH_2Cl_2 via slow evaporation of the solvent (after 5 days suitable crystals were obtained). CCDC 1990060.

Table S4. Crystal data and structure refinement for 18Jux_MM07.

Identification code	18Jux_MM07
Empirical formula	$C_{74}H_{94}$
Formula weight	983.49
Temperature/K	153.00(14)
Crystal system	monoclinic
Space group	I2/a
a/Å	31.8102(6)
b/Å	16.4474(4)
c/Å	26.8541(5)
α/°	90
β/°	98.6448(18)
γ/°	90
Volume/Å^3	13890.3(5)
Z	8
ρ_{calc}g/cm^3	0.941
μ/mm^{-1}	0.387
F(000)	4304.0
Crystal size/mm^3	0.62 × 0.092 × 0.053
Radiation	CuKα (λ = 1.54184)
2Θ range for data collection/°	5.62 to 129.546
Index ranges	-36 ≤ h ≤ 29, -11 ≤ k ≤ 19, -31 ≤ l ≤ 30
Reflections collected	22253
Independent reflections	11273 [R_{int} = 0.0315, R_{sigma} = 0.0331]
Data/restraints/parameters	11273/10/692
Goodness-of-fit on F^2	1.088
Final R indexes [I>=2σ (I)]	R_1 = 0.0665, wR_2 = 0.1767
Final R indexes [all data]	R_1 = 0.0793, wR_2 = 0.1952
Largest diff. peak/hole / e Å^{-3}	0.68/-0.4
CCDC No.	1990060

HBC-based-[5]-helicene 7

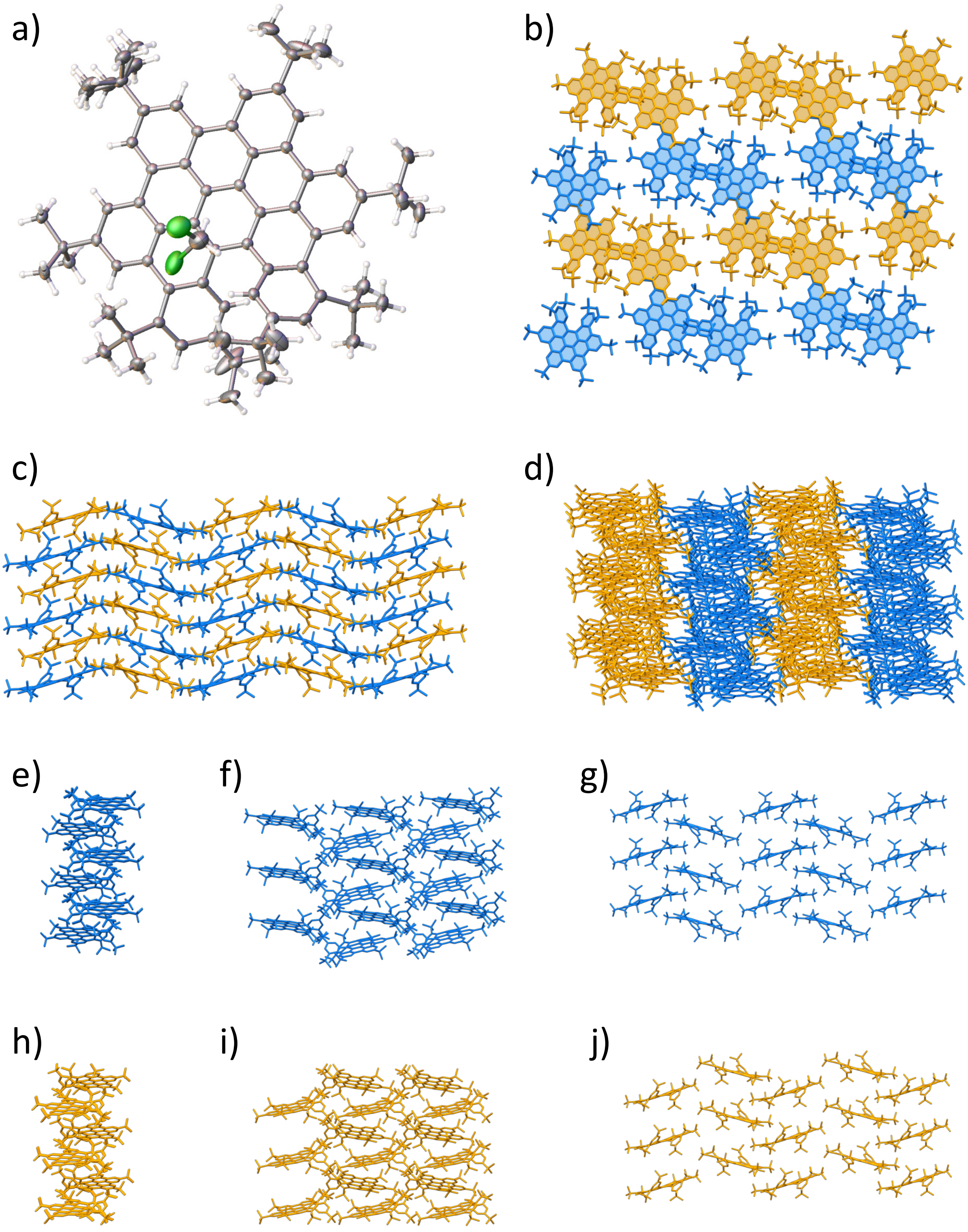

Figure S5. Crystal structure of HBC-based-[5]-helicene **7**. a) ORTEP representation with thermal ellipsoids drawn at 50 % probability; b) – d) packing motif; e) – j) part of the packing motif with molecules of only one chirality (molecules with the same chirality are drawn in the same color). Crystals were grown in a CH_2Cl_2/MeOH solvent mixture and already obtained after one day. CCDC 1990061.

Table S5. Crystal data and structure refinement for 18Jux_MM08.

Identification code	18Jux_MM08
Empirical formula	$C_{75}H_{86}Cl_2$
Formula weight	1058.33
Temperature/K	153.2(5)
Crystal system	monoclinic
Space group	$P2_1/n$
a/Å	25.8586(5)
b/Å	9.4933(2)
c/Å	26.1639(4)
α/°	90
β/°	94.548(2)
γ/°	90
Volume/Å^3	6402.6(2)
Z	4
ρ_{calc}g/cm^3	1.098
μ/mm^{-1}	1.204
F(000)	2280.0
Crystal size/mm^3	0.585 × 0.288 × 0.091
Radiation	CuKα (λ = 1.54184)
2Θ range for data collection/°	6.778 to 129.398
Index ranges	-29 ≤ h ≤ 29, -10 ≤ k ≤ 10, -21 ≤ l ≤ 30
Reflections collected	35919
Independent reflections	10575 [R_{int} = 0.0351, R_{sigma} = 0.0266]
Data/restraints/parameters	10575/15/719
Goodness-of-fit on F^2	1.028
Final R indexes [I>=2σ (I)]	R_1 = 0.0753, wR_2 = 0.2157
Final R indexes [all data]	R_1 = 0.0855, wR_2 = 0.2287
Largest diff. peak/hole / e Å^{-3}	0.91/-0.87
CCDC No.	1990061

4 Comparison to Literature Known [5]Helicenes

Table S6. Comparison of a selection of [5]helicenes. Crystallographic data was obtained free of charge from The Cambridge Crystallographic Data Centre.[10]

	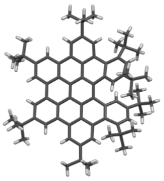	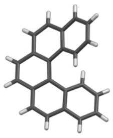	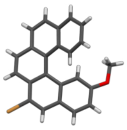	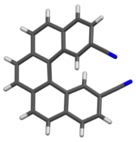	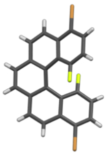	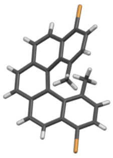
sum of torsion angles	81.5°	65.9 – 67.8°	63.3°	63.9°	70.5 – 72.3°	83.6°
interplanar angle	42.2°	47.3 – 51.3°	45.1°	40.9°	48.0 – 49.5°	48.0°
CCDC	1990061	933556	901873	101630	1528975	1528976
reference	/	Chem. Eur. J. 2013, 19, 16295 – 16302	J. Org. Chem. 2015, 80, 6502 – 6508	Eur. J. Org. Chem. 1999, 1709 – 1718	Org. Lett. 2017, 19, 3707 – 3710	Org. Lett. 2017, 19, 3707 – 3710

Table S7. Comparison of a selection of π-extended [5]helicenes. Crystallographic data was obtained free of charge from The Cambridge Crystallographic Data Centre.[10] The structures with no CCDC number were obtained free of charge from http://pubs.acs.org.

	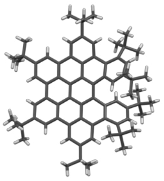	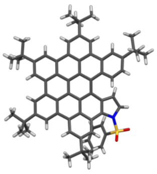	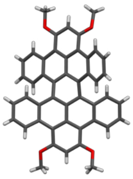	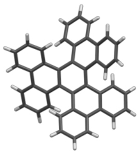	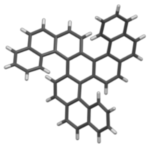	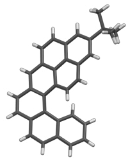
sum of torsion angles	81.5°	67.6°	73.2 – 79.5°	75.7 – 80.7°	68.9 – 71.8°	61.7°
interplanar angle	42.2°	50.3°	59.0 – 71.6°	60.2 - 65.3°	51.3 – 53.7°	44.1°
CCDC	1990061	1527065	/	/	1499672	933555
reference	/	Org. Chem. Front., 2017,4, 861 – 870	J. Am. Chem. Soc. 2012, 134, 13796 – 13803	J. Am. Chem. Soc. 1999, 121, 727 – 733	J. Org. Chem. 2017, 82, 5663 – 5668	Chem. Eur. J. 2013, 19, 16295 – 16302

5 Spectral Appendix

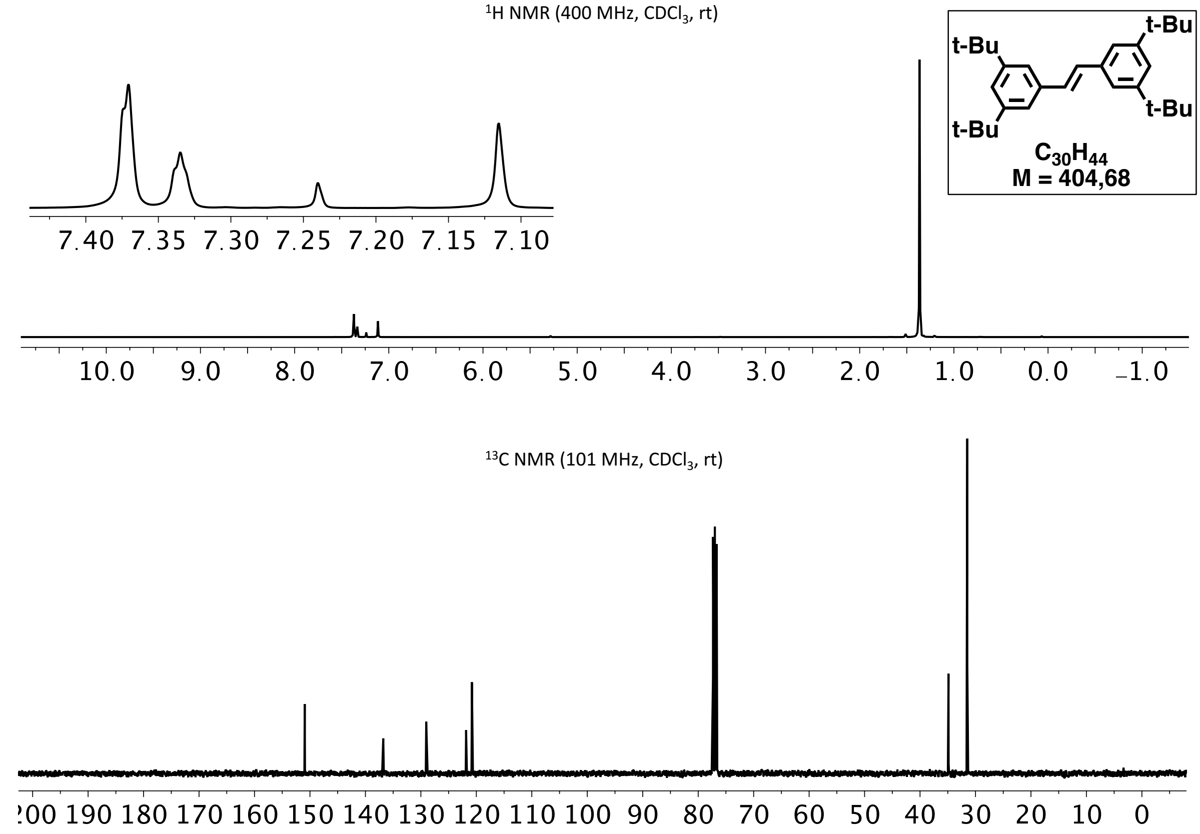

Figure S6. ^{1}H and ^{13}C NMR spectra of 1,2-bis(3,5-di-tert-butylphenyl)ethylene **2**.

HRMS (APPI)

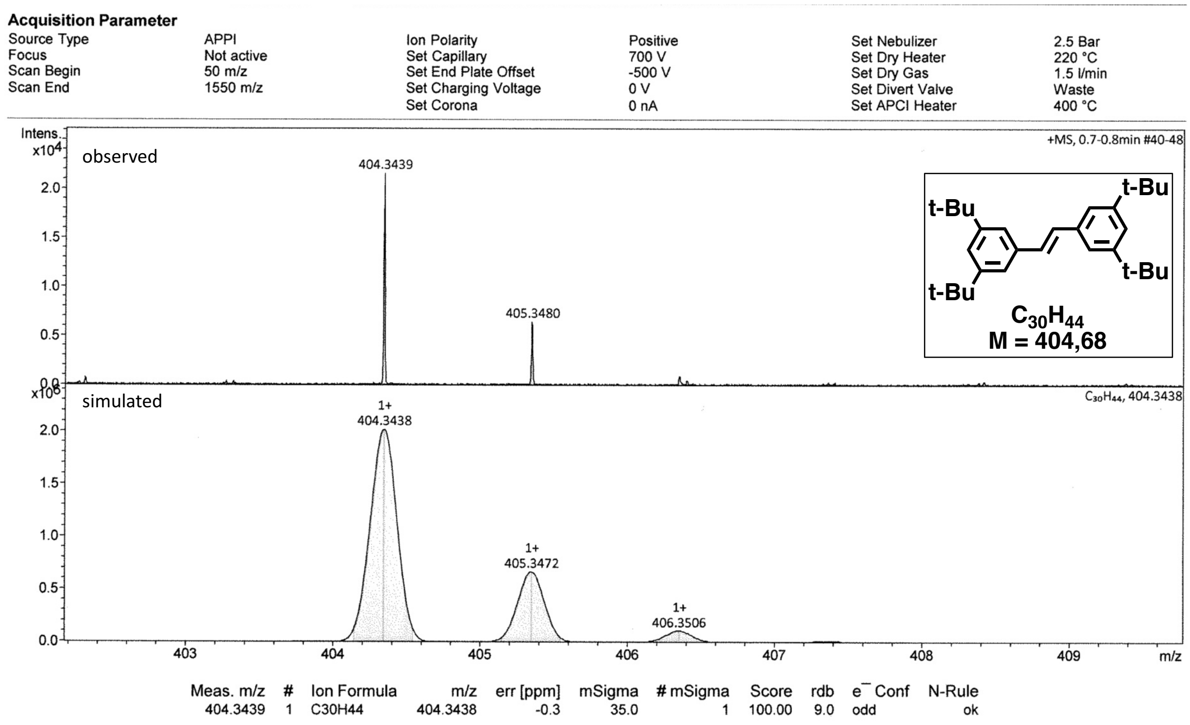

Figure S7. HRMS (APPI) of 1,2-bis(3,5-di-tert-butylphenyl)ethylene **2**.

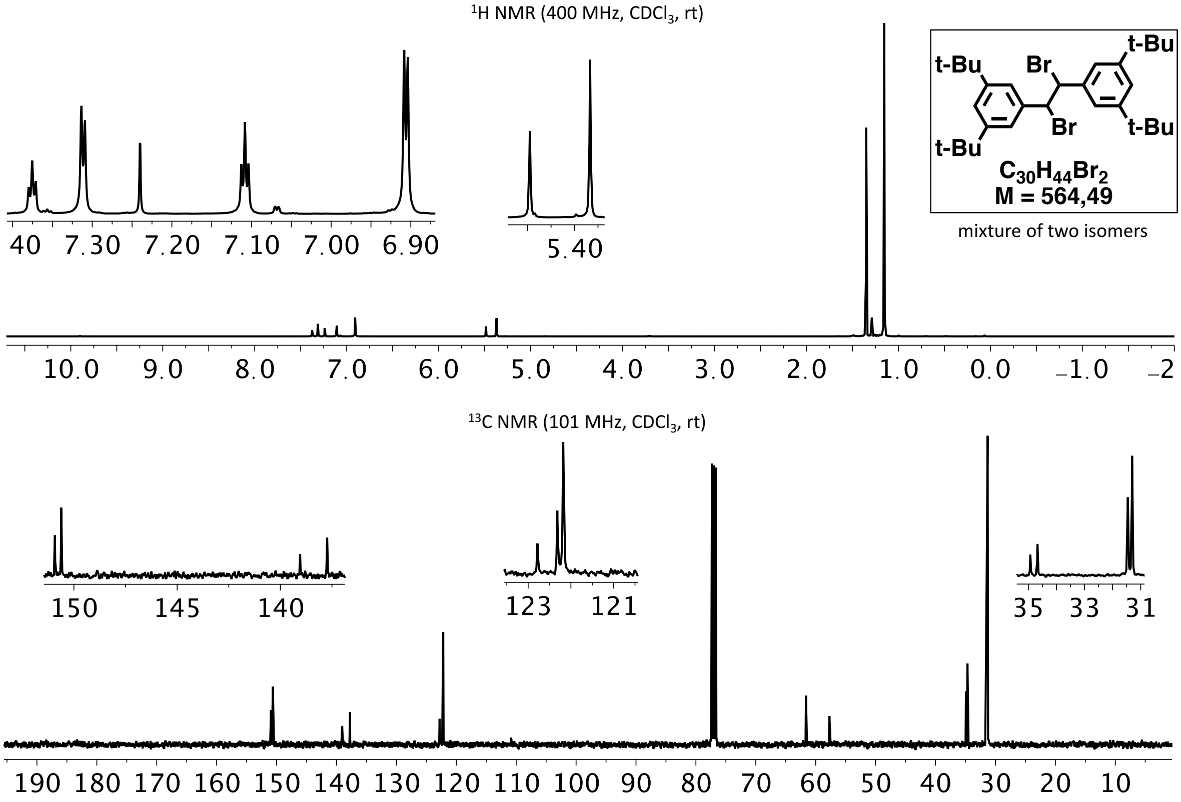

Figure S8. ^{1}H and ^{13}C NMR spectra of **3**.

HRMS (APPI)

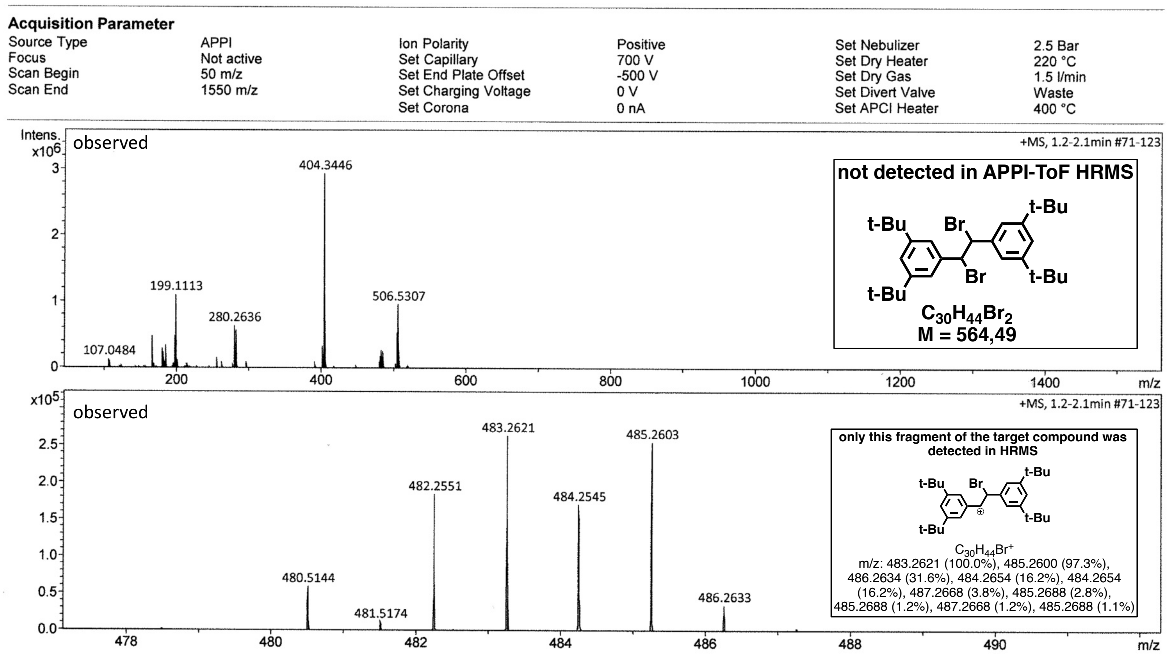

Figure S9. HRMS (APPI) of **3** (product could not be detected).

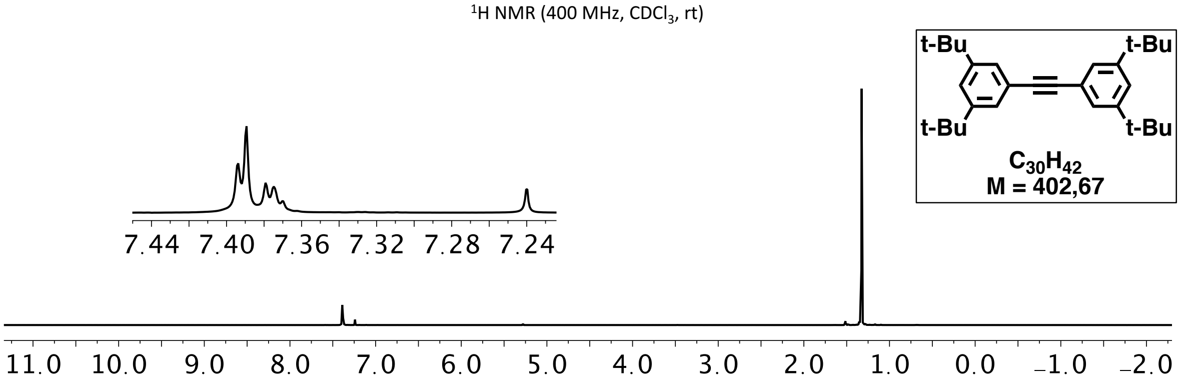

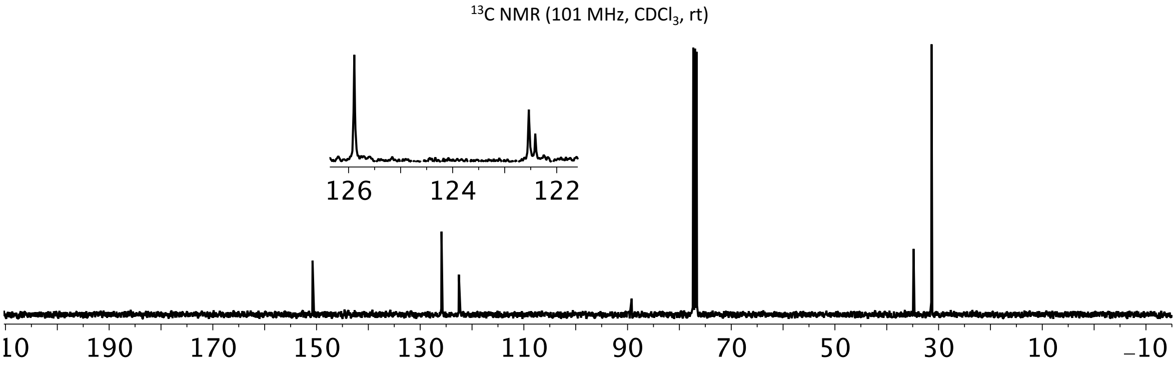

Figure S10. ^{1}H and ^{13}C NMR spectra of tetra-*tert*-butyl-tolane **4**.

HRMS (APPI)

Acquisition Parameter

Source Type	APPI	Ion Polarity	Positive	Set Nebulizer	2.5 Bar
Focus	Not active	Set Capillary	700 V	Set Dry Heater	220 °C
Scan Begin	50 m/z	Set End Plate Offset	-500 V	Set Dry Gas	1.5 l/min
Scan End	1550 m/z	Set Charging Voltage	0 V	Set Divert Valve	Waste
		Set Corona	0 nA	Set APCI Heater	400 °C

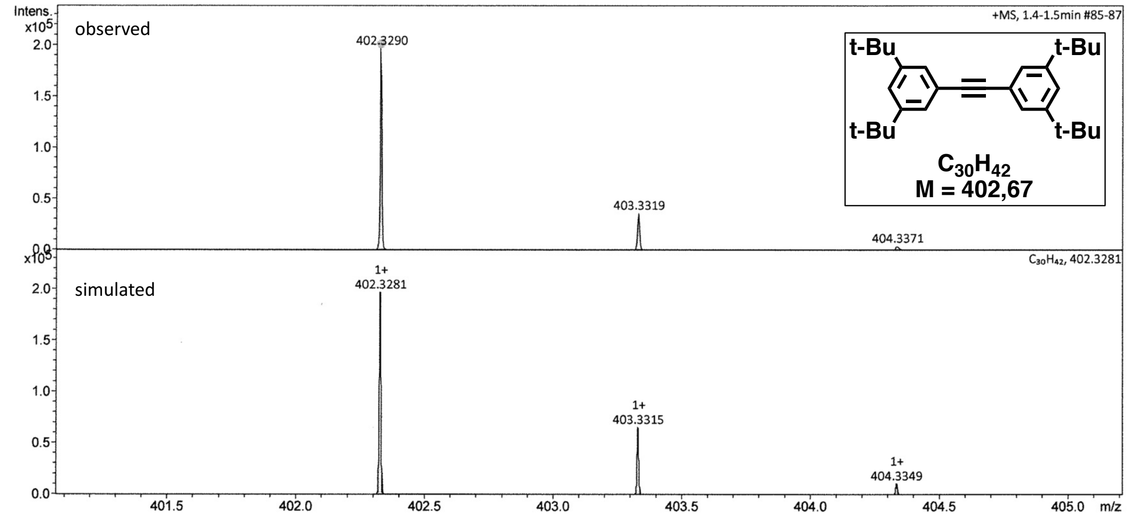

Meas. m/z	#	Ion Formula	m/z	err [ppm]	mSigma	# mSigma	Score	rdb	e⁻ Conf	N-Rule
402.3290	1	C13H36N15	402.3273	-4.4	10.9	1	51.63	3.5	even	ok
	2	C15H38N12O	402.3286	-1.1	17.7	2	100.00	3.0	odd	ok
	3	C17H40N9O2	402.3299	2.3	24.6	3	67.69	2.5	even	ok
	4	C30H42	402.3281	-2.3	88.1	4	8.34	10.0	odd	ok

Figure S11. HRMS (APPI) of tetra-*tert*-butyl-tolane **4**.

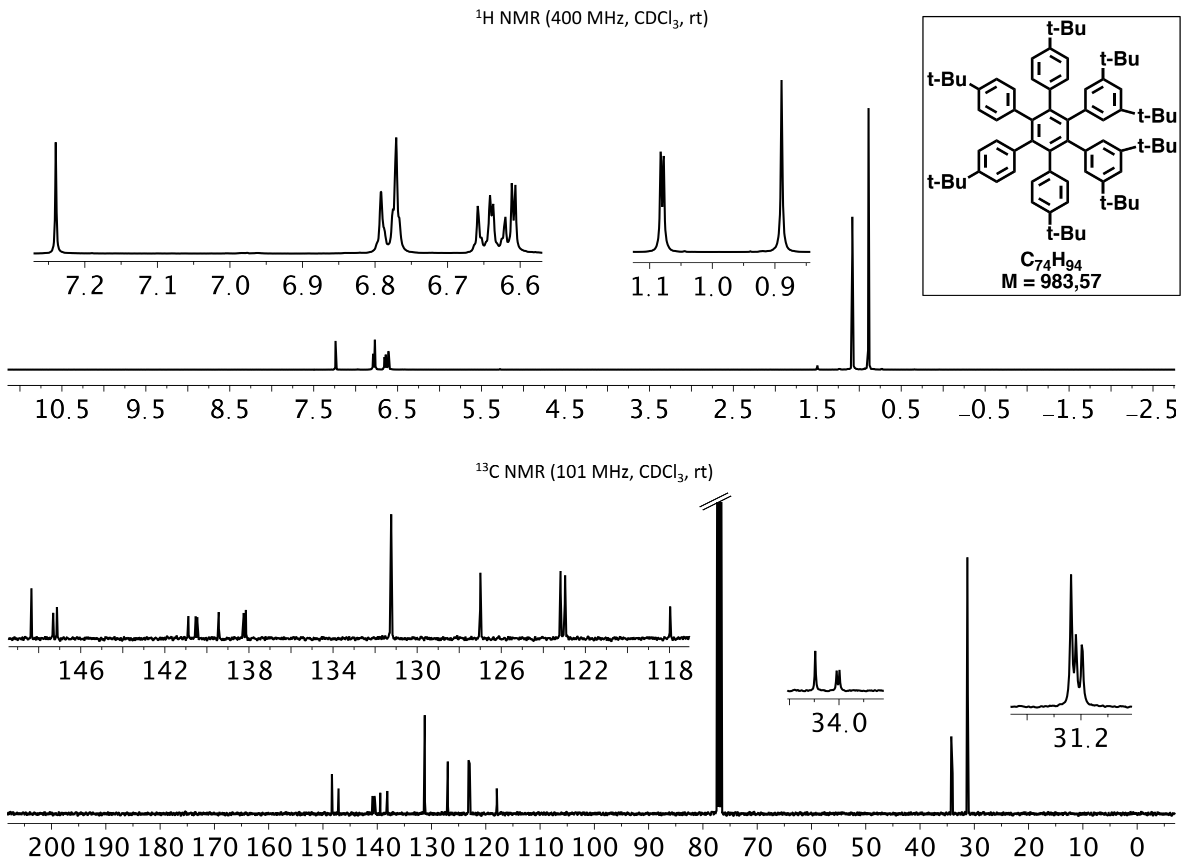

Figure S12. ^{1}H and ^{13}C NMR spectra of octa-*tert*-butyl-HPB **6**.

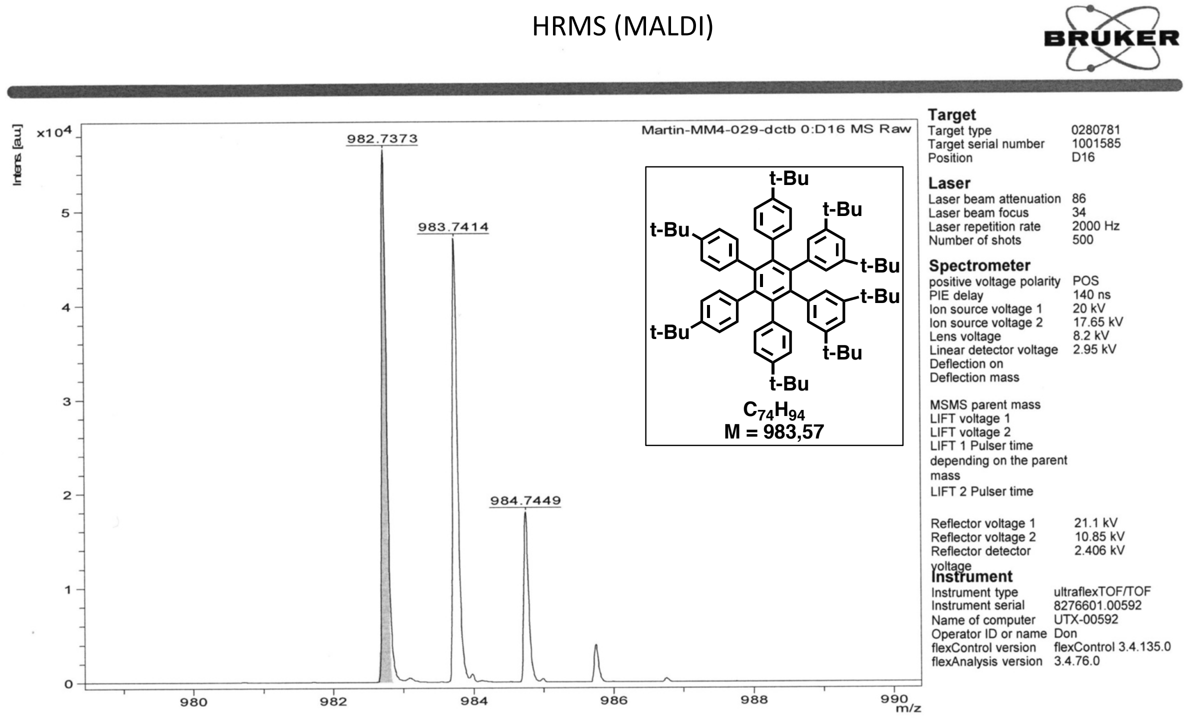

SmartFormula

Formula	Mass	Error	mSigma	DblEq	N rule	Electron Configuration
C 74 H 94	982.7350	2.3432	40.4033	28.00	ok	odd

Figure S13. HRMS (MALDI) of octa-*tert*-butyl-HPB **6**.

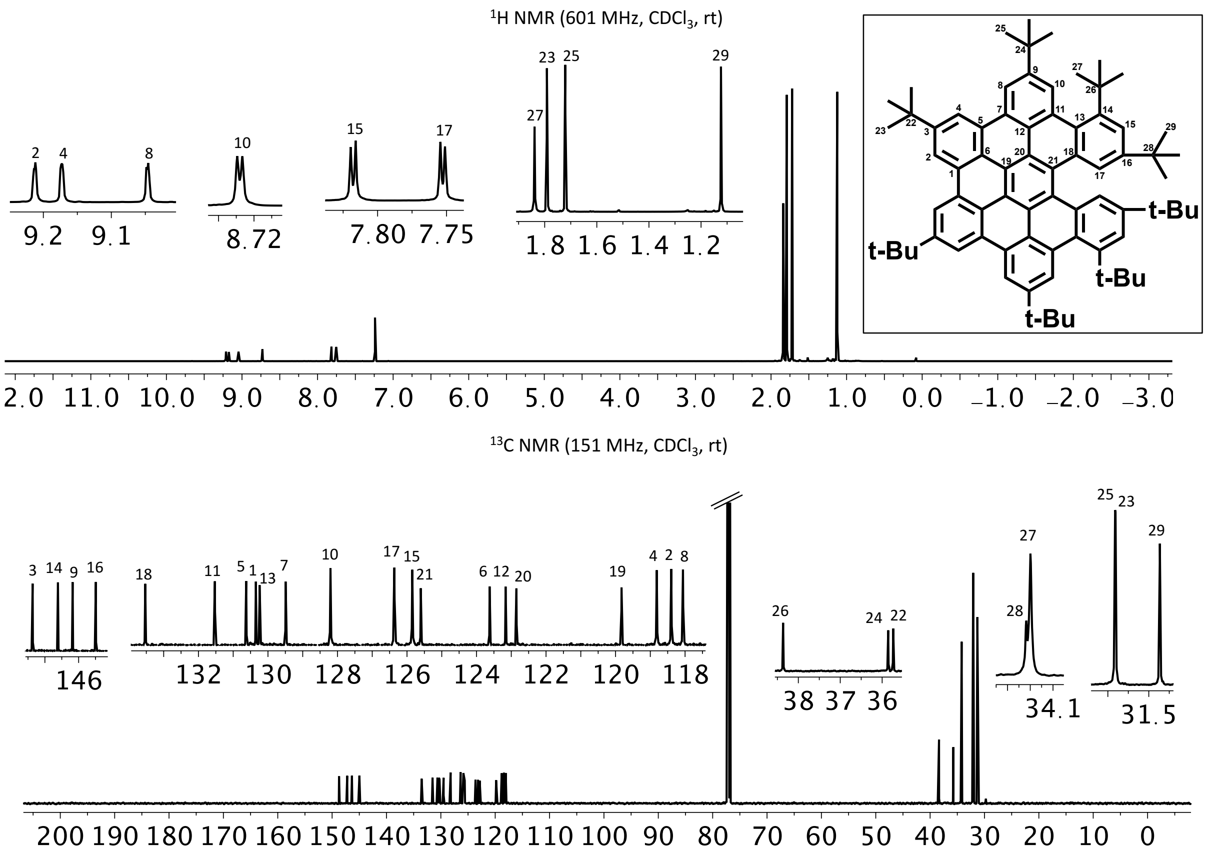

Figure S14. ^{1}H and ^{13}C NMR spectra of HBC-based-[5]-helicene **7**. Signals were assigned with the help of DEPTq135, COSY, HSQC, HMBC and NOE experiments.

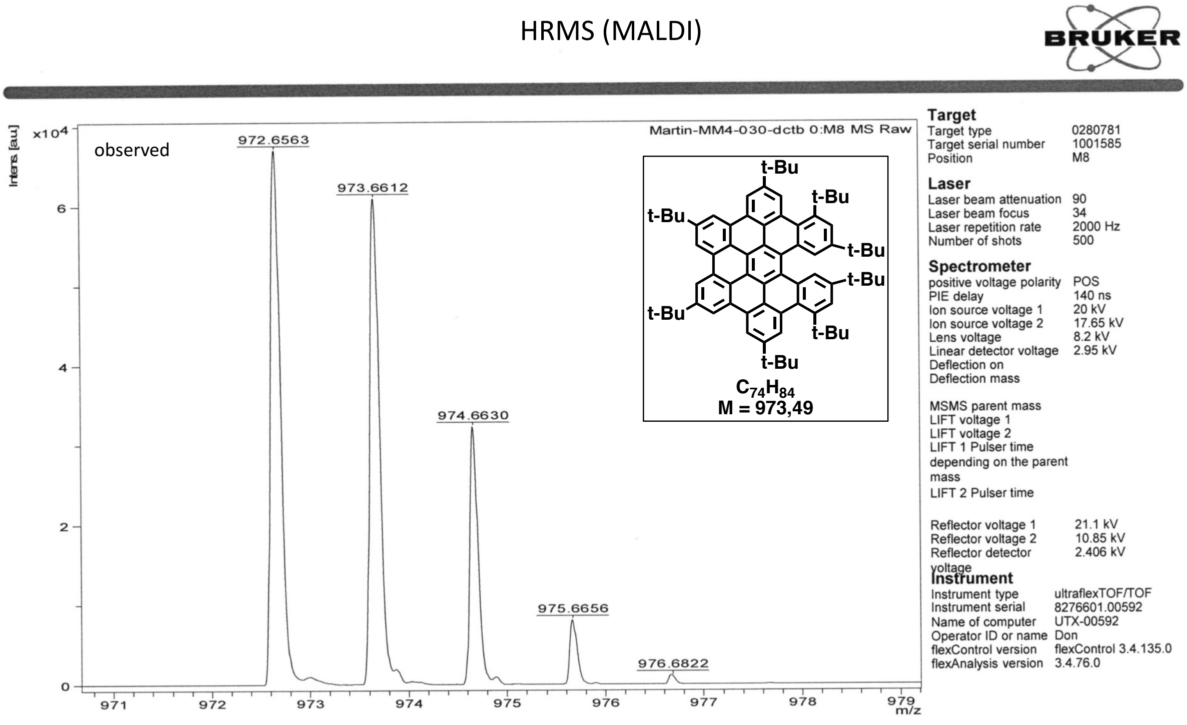

SmartFormula

Formula	Mass	Error	mSigma	DblEq	N rule	Electron Configuration
C 74 H 84	972.6568	0.4655	85.2682	33.00	ok	odd

Figure S15. HRMS (MALDI) of HBC-based-[5]-helicene **7**.

6 References

[1] M. M. Martin, D. Lungerich, P. Haines, F. Hampel, N. Jux, *Angew. Chem. Int. Ed.* **2019**, *58*, 8932-8937.

[2] D. Reger, P. Haines, F. W. Heinemann, D. M. Guldi, N. Jux, *Angew. Chem. Int. Ed.* **2018**, *57*, 5938-5942.

[3] K. S. Jeong, Y. S. Kim, Y. J. Kim, E. Lee, J. H. Yoon, W. H. Park, Y. W. Park, S. J. Jeon, Z. H. Kim, J. Kim, N. Jeong, *Angew. Chem. Int. Ed.* **2006**, *45*, 8134-8138.

[4] G. Westman, O. Wennerström, I. Raston, *Tetrahedron* **1993**, *49*, 483-488.

[5] K. Ishimaru, K. Monda, Y. Yamamoto, K.-Y. Akiba, *Synth. Commun.* **2000**, *30*, 575-580.

[6] Y. Qi, Y. Luan, J. Yu, X. Peng, G. Wang, *Chemistry* **2015**, *21*, 1589-1597.

[7] H. G. Aurich, J. Eidel, M. Schmidt, *Chem. Ber.* **1986**, *119*, 18-35.

[8] L. Ackermann, A. V. Lygin, N. Hofmann, *Org. Lett.* **2011**, *13*, 3278-3281.

[9] H.-P. Jacquot de Rouville, R. Garbage, R. E. Cook, A. R. Pujol, A. M. Sirven, G. Rapenne, *Chem. Eur. J.* **2012**, *18*, 3023-3031.

[10] C. R. Groom, I. J. Bruno, M. P. Lightfoot, S. C. Ward, *Acta Cryst. B* **2016**, *72*, 171-179.
